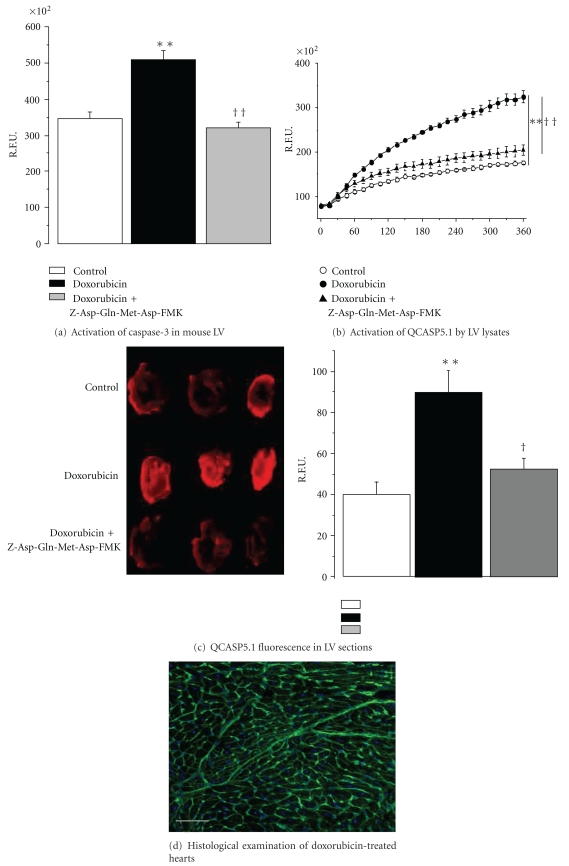# *In Vitro* and *Ex Vivo* Evaluation of Smart Infra-Red Fluorescent Caspase-3 Probes for Molecular Imaging of Cardiovascular Apoptosis

**DOI:** 10.1155/2011/413290

**Published:** 2011-05-05

**Authors:** Manuelle Debunne, Christophe Portal, Bruno Delest, Ebba Brakenhielm, Françoise Lallemand, Jean-Paul Henry, Heidi Ligeret, Pauline Noack, Marc Massonneau, Anthony Romieu, Pierre-Yves Renard, Christian Thuillez, Vincent Richard

**Affiliations:** ^1^Inserm U644 and Rouen University Hospital, Institute for Biomedical Research and IRFMP23, University of Rouen, 76183 Rouen, France; ^2^QUIDD, 50 rue Ettore Bugatti, Technopôle du Madrillet, 76800 Saint-Etienne du Rouvray, France; ^3^Equipe de Chimie Bio-Organique, COBRA-CNRS UMR 6014 & FR 3038, rue Lucien Tesnière, 76131 Mont-Saint-Aignan, France; ^4^Department of Chemistry, University of Rouen, Place Emile Blondel, 76821 Mont-Saint-Aiginan, France; ^5^Department of Chemistry, Institut Universitaire de France, 103 Boulevard St. Michel, 75005 Paris, France

## Abstract

*Purpose*. The aim of this paper is to develop new optical bioprobes for the imaging of apoptosis. *Procedure*. We developed quenched near-infrared probes which become fluorescent upon cleavage by caspase-3, the key regulatory enzyme of apoptosis. *Results*. Probes were shown to be selectively cleaved by recombinant caspase-3. Apoptosis of cultured endothelial cells was associated with an increased fluorescent signal for the cleaved probes, which colocalized with caspase-3 and was reduced by the addition of a caspase-3 inhibitor. Flow cytometry demonstrated a similar profile between the cleaved probes and annexin V. *Ex vivo* experiments showed that sections of hearts obtained from mice treated with the proapoptotic drug doxorubicin displayed an increase in the fluorescent signal for the cleaved probes, which was reduced by a caspase-3 inhibitor. *Conclusion*. We demonstrated the capacity of these novel probes to detect apoptosis by optical imaging *in vitro* and *ex vivo*.

## 1. Introduction

Apoptosis, or programmed cell death, is a well-organized mechanism that allows cells to die without leading to inflammation, contrary to necrosis [1, 2]. Apoptosis plays a critical role in a number of physiological functions such as fetal development or tissue homeostasis regulation [3]. In addition to these physiological roles, considerable evidence suggests that changes in apoptosis play a major role in the development of various diseases. Indeed, in cancer, inhibition of apoptosis leads to anarchic division of cells and favors tumor development. In contrast, overactivation of apoptosis contributes to the aggravation of various cardiovascular diseases such as atherosclerosis, myocardial, infarction, and heart failure [4].

Over the past years, many methods have been developed to detect apoptosis, most of them targeting phosphatidylserine (PS) which is normally expressed on the inner plasma membrane. However, in apoptotic conditions, aminophospholipid translocase inhibition and scramblase activation lead to PS translocation to the outer leaflet of the plasma membrane [5, 6]. Thus, PS becomes easily accessible to detect apoptosis [7]. Noninvasive imaging methods targeting PS have been developed. Indeed, natural ligands of this phospholipid, annexin V or the C2A domain of synaptotagmin [8], have been labeled with radioisotopes (^99m^Tc) for single photon emission computed tomography imaging [9, 10], with magnetic nanoparticles for magnetic resonance imaging [11], or with near-infrared fluorescent labels for optical imaging [12].

Despite this wide availability of biomarkers that use PS for imaging of apoptosis, this approach suffers from several limitations. Indeed, necrosis induces degradation of cell membrane and, thus, exposition of PS to labeled annexin V or synaptotagmin, leading to a false positive signal [13]. Because of these limitations of the currently available methods for apoptosis imaging, there is a clear need for new methods based on smart probes that target specific molecular events related to the apoptosis phenomenon.

Two major pathways compose apoptosis. The first one is extrinsic signaling, or death receptor-mediated pathway. The second one is the intrinsic, mitochondrial-dependent pathway, which is activated in response to extracellular stimuli or DNA damage [14]. Both pathways, however, commonly involve activation of cysteinyl-aspartate-specific proteases, or caspases, and especially require activation (by cleavage) of caspase-3 [15, 16]. Because of its central position on major apoptosis pathways, activated caspase-3 is a target of choice for the detection of programmed cell death both *in vitro* and *in vivo* and has already been chosen to image (noncardiac) apoptosis in various models [17–20].

Thus, the purpose of the present study is to develop and validate new FRET-based smart molecular probes for the detection of apoptosis, based on their ability to emit near-infrared fluorescence signal upon selective cleavage by activated caspase-3.

## 2. Material and Methods

### 2.1. *In Vitro* Cleavage of Probes by Recombinant Caspases

Recombinant caspases were incubated in buffer (100 mM NaCl, 40 mM HEPES, 10 mM DTT, 1 mM EDTA, 10% (w/v) sucrose, and 0.1% (w/v) CHAPS, pH 7.4) for 75 min at 37°C. The final concentration of each caspase was adjusted to obtain a volumic activity of 0.5 pmol/min/*μ*L in the well. The caspase-activatable probe was then added (final concentration: 1 *μ*M), and the kinetics of cleavage in each condition was monitored at 37°C for 5 h on the FlexStation II microplate reader: fluorescence emission was recorded at 670 nm every 60 seconds with an excitation wavelength set at 645 nm. In some wells, the caspase-3 and caspase-7 inhibitor Ac-Asp-Glu-Val-Asp-CHO was added at a final concentration of 0.25 *μ*M and incubated at 37°C for 60 min before probes addition.

### 2.2. Cells Line and Culture Conditions

Murine heart microvascular endothelial cells (HME) were cultured in Dulbecco's modified eagle's medium (DMEM) enriched with 4.5 g·L^−1^ glucose and supplemented with 1% glutamine, 10% fetal bovine serum (FBS), 50 units/mL penicillin, and 50 *μ*g/mL streptomycin. Cells were grown at 37°C in a humidified 95% O_2_-5% CO_2_ atmosphere. After being starved from growth factors, using (FBS 2%), cells were incubated with solvent or staurosporine at 1 *μ*M, for 6 h, in order to induce apoptosis [21]. Staurosporine-treated cells were treated or not with the caspase-3 inhibitor (Z-Asp(OMe)-Gln-Met-Asp(OMe)-CH_2_F) which was purchased from Calbiochem (San Diego, CA, USA) [22].

### 2.3. Evaluation of Caspase-3 Activity

Caspase-3 activity was evaluated in HME cells cultured in 96 well plates (around 10000 cells/well) which were seeded and treated as describe above. Cells were then incubated with a caspase 3/7 glow kit (Promega) for 30 min, and the luminescence signal was read using a Mithras Multimode Microplate Reader LB 940 (Berthold Tech, Thoiry, France).

### 2.4. Evaluation of Probe Fluorescence in Cell Lysates

HME cells seeded in a 10 cm Petri dish were scrapped in cold caspase-3 buffer and incubated with the probe (1 *μ*M) at 37°C. The fluorescence signal was then followed over 6 h using a FlexStation II (Bucher Biotec).

### 2.5. Evaluation of Probes Penetration in Cells

HME cells seeded in 6 well-dishes were incubated with the probe for 1 hour, in caspase-3 buffer, after which the supernatant (cell medium) was removed, and the cells were trypsinised and lysed. Both the cell medium and the cell lysates were incubated overnight with recombinant caspase-3 (0.5 pmol/min/*μ*L) at 37°C. Fluorescence was read with a fluorescent microplate spectrometer. The penetration of probes was assessed using the ratio of cell/medium fluorescence.

### 2.6. Flow Cytometry Analysis

Flow cytometry analysis of the probes or annexin V was performed in HME cells seeded in a 10 cm Petri dish. Then, cells were trypsinized and incubated with 10 *μ*L/mL annexin V (Beckton Dickinson) or 1 *μ*M probe for 15 min at room temperature in a dark atmosphere. Analysis was carried out using a Becton Dickinson FACScan flow cytometer (Mountain View, CA). Results were analyzed using FlowJo software (Tree Star, Ashland, USA). Only cells with an FL4 fluorescence intensity between 10^2^ to 10^4^ were included in the analysis gate.

### 2.7. Fluorescence Microscopy

HME cells were cultured in 8-well slide chambers (Labtek; Becton Dickinson, Lane Cove, New South Wales, Australia). After treatment, cells were rinsed with phosphate buffered saline (PBS) 1X, followed by fixation with cold methanol for 10 min at −20°C. Cells were then saturated in PBS 1X/bovine serum albumin 1%, overnight, after which they were rapidly washed with PBS 1X and incubated with a rabbit mAb against cleaved (activated) caspase-3 (1 : 100, cell signaling) at room temperature for 1 h. After washing with PBS 1X, cells were incubated with a Cy 3.0-labeled donkey antirabbit IgG Ab (1 : 100; Jackson Immunoresearch) and with Bisbenzimide H33342 (Hoechst, Riedel-de Haën) at 0.1 *μ*g/mL for 2 min. In some experiments, slides were also incubated with 1 *μ*M probe for 2 h at 37°C. Coverslips were rapidly rinsed and mounted in VECTASHIELD fluomounting medium (Vector) and observed using an Axio Imager Z.1 fluorescence microscope equipped with ApoTome technology (Zeiss, Oberkochem, Germany).

### 2.8. Animal Model of Apoptosis

Cardiac apoptosis induced by doxorubicin (DOX) as previously described. [23, 24]. For this purpose, 8-week-old C57BL6/J male mice (Janvier) received an intraperitoneal injection of 20 mg/kg DOX (Sigma). Four days later, mice were euthanized by cervical dislocation, the heart was excised, and the left ventricle (LV) was frozen until analysis. These *in vivo* experiments followed the institutional and national guide for the care and use of laboratory animals.

In order to assess the possible effects of the treatment on cell integrity, 4 *μ*M section were obtained from frozen LV using a cryotome. Sections were fixed in acetone for 30 s then rinsed in PBS 1X. Structure integrity was assessed with 5.0 *μ*g/mL of wheat germ agglutine conjugated with fluorescein (Invitrogen) for 30 min. Nuclei were counterstained with Bisbenzimide H33342 (Hoechst Riedel de Haën) at 0.1 *μ*g/mL for 2 min. Sections were analyzed using an Axio Imager Z.1 fluorescence microscope equipped with ApoTome technology (Zeiss, Oberkochem, Germany).

### 2.9. Evaluation of Caspase-3 Activity in Mouse LV

Frozen LV were homogenized in 200 *μ*L ice-cold caspase-3 buffer, and homogenates were then centrifuged for 5 min, at 12000 rpm, and the amount of proteins present in the supernatant was evaluated using a Bradford assay. All samples were then diluted to 2 mg of protein/mL, incubated for 30 min with the same volume of caspase 3/7 glow kit, and the luminescence signal was read using a Mithras Multimode Microplate Reader LB 940.

### 2.10. Evaluation of Probes Fluorescence in Mouse LV Lysates

Frozen LV were homogenized and diluted as describe above. Samples were then incubated with 5 *μ*M probe at 37°C, and the fluorescence signal was followed over 6 h, using a FlexStation II (Bucher Biotec).

### 2.11. Evaluation of Probes Fluorescence in Mouse Thick LV Sections

Fresh LV was sliced in 1 mm sections and incubated with PBS or with the caspase-3 inhibitor Z-Asp(OMe)-Gln-Met-Asp(OMe)-CH_2_F (100 *μ*M) for 30 min, after which the sections were incubated with the probe (1 *μ*M) for 1 h. Fluorescence of the slices was analyzed using an infrared imaging system, Odissey (LI-COR Bioscience).

### 2.12. Colocalization of Probe Fluorescence with Caspsase-3 in LV Sections

Frozen LV were sliced in 4 *μ*m section using a cryotome. Sections were fixed in acetone for 30 s then rinsed in PBS 1X. Fluorescence emitted by the cleaved probe as well as that of the anticaspase-3 antibody were analyzed as described above for cultured cells.

### 2.13. Statistical Analysis

Results were expressed as means ± SEM. Data were analyzed using either Student's *t*-test or by one-way or two-way ANOVA, eventually followed by Student's *t*-test with Bonferroni correction. A *P* value <.05 was considered to be statistically significant.

## 3. Results

### 3.1. Validation of the *In Vitro* Model of Apoptosis

**Figure 1:**
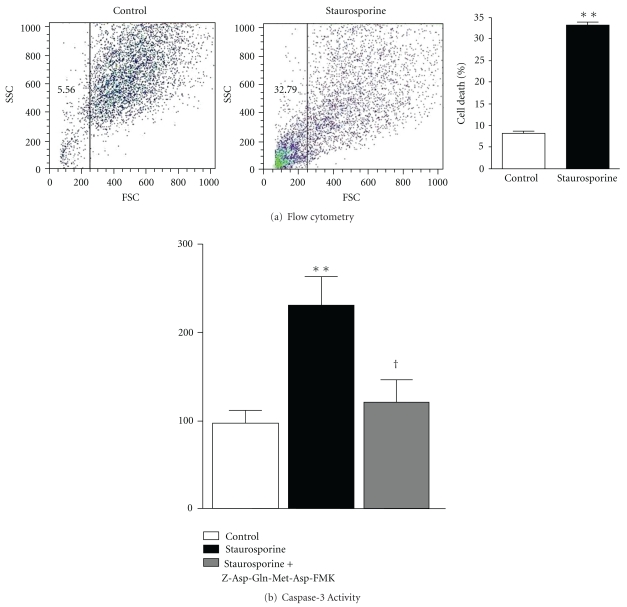
(a) Typical flow cytometry signal obtained in HME cells. Cells were either untreated (control) or incubated with staurosporine 1 *μ*M for 6 h, and percentage of cell death, derived from the flow cytometry analysis, obtained in control or staurosporine-treated HME cells. Data are mean ± SEM (*n* = 30). (b) Caspase-3 activity in HME cells. Cells were either untreated (control) or treated with staurosporine in the absence or the presence of the caspase-3 inhibitor Z-Asp(OMe)-Gln-Met-Asp(OMe)-CH_2_F (100 *μ*M). R.L.U.: relative luminescent units. Data are mean ± SEM (*n* = 6). **P < .01 versus controls; ^†^ *P* < .05 versus staurosporine alone.

Apoptosis was induced *in vitro* by incubating heart microvascular endothelial (HME) cells with 1 *μ*M staurosporine for 6 h. Cell death, assessed by flow cytometry analysis, is shown in Figure 1. Compared to control cells, staurosporine-treated cells displayed a flow cytometry profile characteristic of increased cell death (Figure 1(a)). Quantitative analysis of the scatter plot revealed that staurosporine markedly (almost 6 times) and significantly increased cell mortality (Figure 1(a)).  Figure 1(b) shows that staurosporine more than doubled caspase-3 activity compared to control cells. Moreover, incubation of the cells with Z-Asp(OMe)-Gln-Met-Asp(OMe)-fluoromethylketone (Z-Asp(OMe)-Gln-Met-Asp(OMe)-CH_2_F), a specific inhibitor of caspase-3, before staurosporine treatment, reduced caspase-3 activity, which became not significantly different from controls.

### 3.2. Evaluation of the Cleavage Selectivity of QCASP3.2 towards Recombinant Enzymes

**Figure 2:**
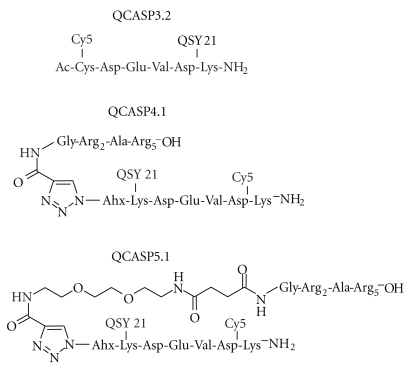
Chemical structure of QCASP3.2, QCASP4.1, and QCASP5.1.

**Figure 3:**
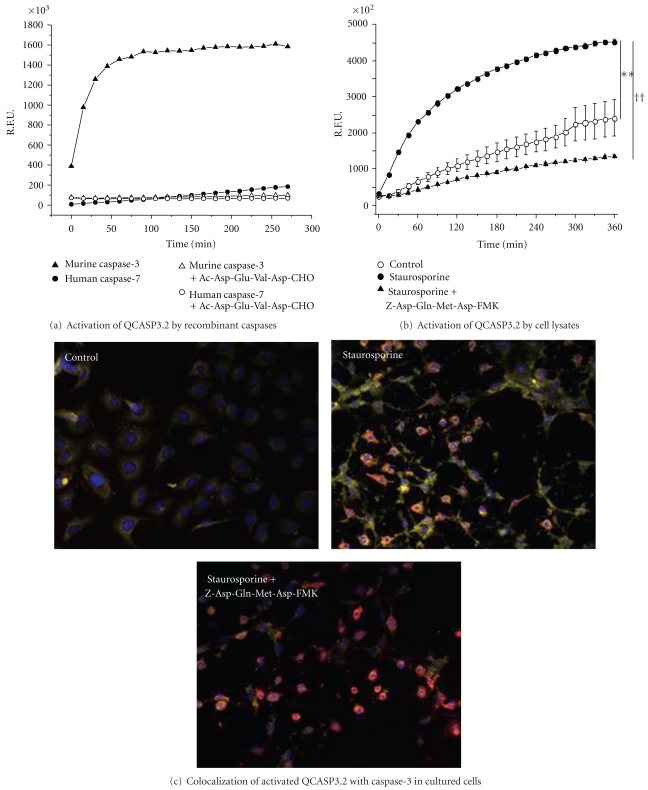
(a) Activation of QCASP3.2 by recombinant caspases. R.F.U.: relative fluorescent units. Cleavage by caspases 3 and 7 in the absence or presence of the caspase inhibitor Ac-Asp-Glu-Val-Asp-CHO (250 nM) is shown. No fluorescence was detected with caspases 1, 6, 8, and 11, thus these data are not presented. (b) Activation of QCASP3.2 by lysates of HME cells. Cells were either untreated (control) or treated with staurosporine 1 *μ*M for 6 h, in the absence or the presence of the caspase-3 inhibitor Z-Asp(OMe)-Gln-Met-Asp(OMe)-CH_2_F (100 *μ*M). R.F.U.: relative fluorescent units. Data are mean ± SEM (*n* = 3). **P < .01 versus control and ^††^ *P* < .01 versus staurosporine alone by repeated measures ANOVA. (c) Colocalization of activated QCASP3.2-induced fluorescence (yellow) with caspase-3 IF (red) in HME cells. Cells were treated or not with staurosporine 1 *μ*M for 6 hours, in the absence or the presence of the caspase-3 inhibitor Z-Asp(OMe)-Gln-Met-Asp(OMe)-CH_2_F (100 *μ*M). Nuclei are stained with Hoechst and appear blue. Orange staining is the colocalization of QCASP3.2 (yellow) with caspase-3 mAb (red; magnification x20).

QCASP3.2 consists of the well-known Asp-Glu-Val-Asp caspase-3 tetrapeptide substrate flanked on its N and C termini, respectively, by a Cysteine and a Lysine, allowing for the attachment of a Cy5.0 dye and a QSY21 quencher (Figure 2). Specificity of QCASP3.2 was studied by assessing its activation in a presence of a panel of recombinant active caspases (1, 3, 6, 7, 8, and 11). QCASP3.2 is cleaved by caspase-3 and, to a very small extent, by caspase-7, and both cleavages were suppressed by the caspase-3/caspase 7 inhibitor Ac-Asp-Glu-Val-Asp-CHO (Figure 3(a)). In contrast, no detectable cleavage was observed in the presence of caspases 1, 6, 8, and 11 (data not shown).

### 3.3. Evaluation of the Capacity of QCASP3.2 to Detect Caspase-3 Activation *In Vitro*

The fluorescence generated by the cleavage of QCASP3.2 (1 *μ*M) incubated with apoptotic cell lysates over 6 h is shown in Figure 3(b). Compared to control cells, staurosporine-treated cells presented an almost doubled fluorescence level (at 6 h: control 2418 ± 508; staurosporine 4532 ± 736 R.F.U. × 10^2^; *P* < .01). This increased fluorescence was abolished by a pretreatment with the selective caspase-3 inhibitor Z-Asp(OMe)-Gln-Met-Asp(OMe)-CH_2_F. Indeed, in the presence of the inhibitor, fluorescence at 6 h was reduced to 1357 ± 402 R.F.U. × 10^2^ (*P* < .01 versus staurosporine alone).

Results on the activation of QCASP3.2 and on the colocalization with caspase-3 in cultured cells are presented in Figure 3(c). In control cells, in which little or no activated caspase-3 staining (red) was observed, we found low activation of QCASP3.2 (yellow). 

In contrast to controls, in staurosporine-treated cells, the increased positive IF staining for activated caspase-3 was accompanied by a strong increase in the fluorescence signal emitted by the cleaved QCASP3.2 probe, which was partly reduced by the caspase-3 inhibitor. Moreover, in those cells, we observed a colocalization of the antiactivated caspase-3 mAb and cleaved QCASP3.2 staining. Additionally, high resolution examination of the slices revealed that the fluorescence was intracellular and not membrane-bound, although this might only partially reflect the cell penetration in this situation because of the experimental conditions involving methanol fixation of the sections.

Despite the fact that QCASP3.2 brought several promising results in terms of capacity to be cleaved and to become highly fluorescent in the presence of activated caspase-3 (either recombinant or endogenous), this probe did not show satisfactory cell labeling ability in flow cytometry (data not shown). As this is most likely due to insufficient internalization of the probe into cells, we developed second-generation probes, namely, QCASP4.1 and QCASP5.1 (Figure 2), characterized by the addition of a cell-penetrating peptide (CPP) to improve the capacity of the probes to penetrate into cells, and in the case of QCASP5.1, an additional PEG moiety aimed at increasing peptide half-life for future subsequent *in vivo* studies.

### 3.4. Comparison of the Cell-Penetrating Properties of QCASP3.2, QCASP4.1, and QCASP5.1

In order to assess the capacity of the probes to penetrate into cells, HME cells were incubated for 1 h with each probe, after which the cell culture medium (corresponding to the extracellular compartment) was removed, and the cells were lysed (the resulting lysate representing the intracellular compartment).

**Figure 4:**
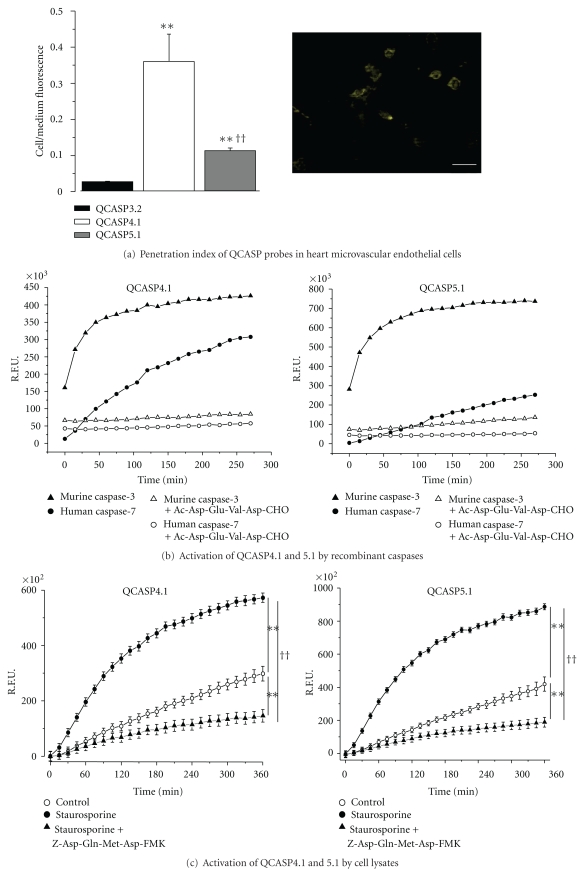


Figure 4(a) (left: penetration index of QCASP probes in HME cells. The penetration index was defined as the ratio of fluorescence measured in the cell lysates over that measured in the cell medium. Data are mean ± SEM (*n* = 12). **P < .01 versus QCASP3.2; ^††^ *P* < .01 versus QCASP4.1; right: representative image of internalization of QCASP5.1 in endothelial cells. The scale bar represents a 20-micrometer length) shows that QCASP4.1 has the highest capacity to penetrate the cells, followed by QCASP5.1 and QCASP3.2. Indeed, compared to QCASP3.2, the ratio cell lysate/cell medium fluorescence was increased 15 and 5 times for QCASP4.1 and QCASP5.1, respectively. A representative image of internalization of QCASP5.1 in endothelial cells is shown in Figure 4(a).

### 3.5. Evaluation of the Cleavage Selectivity of QCASP4.1 and QCASP5.1 towards Recombinant Enzymes (Figure 4(b))

Recombinant caspase-3 assays were performed similarly to QCASP3.2 with the second-generation probes and showed an expected increase of fluorescence as a function of time (Figure 4(b): activation of QCASP4.1 and QCASP5.1 by recombinant caspases. R.F.U.: relative fluorescent units. Cleavage by caspases 3 and 7, with or without the caspase-3/7 inhibitor Ac-Asp-Glu-Val-Asp-CHO (250 nM) is shown. No increased fluorescence was detected either in the presence of caspases 1, 6, 8, and 11, thus these data are not represented on the figure). However, the final level of fluorescence (i.e., after reaching steady state) was lower with both cell penetrating probes QCASP4.1 and QCASP5.1 than with QCASP3.2. Despite this, QCASP4.1 and QCASP5.1 were found to be efficiently cleaved by the target enzyme. In fact, results obtained with cleavage of QCASP4.1 and QCASP5.1 by a series of recombinant caspases (1, 3, 6, 7, 8, and 11) demonstrated a significant cleavage only observed in the presence of caspases 3 and 7 (Figure 4(b)), and no cleavage induced by caspases 1, 6, 8, and 11 (data not shown). In parallel we also found an abolition of the cleavage induced by caspase 3 or 7 in the presence of the nonselective caspase inhibitor Ac-Asp-Glu-Val-Asp-CHO.

### 3.6. Evaluation of the Capacity of QCASP4.1 and QCASP5.1 to Detect Endogenous Caspase-3 Activity *In Vitro*

The capacity of QCASP4.1 and QCASP5.1 to detect caspase-3 activity was also assessed in staurosporine-treated HME cells. Specifically, we analyzed the kinetics of probe activation by cell lysates, and the colocalization of the probe with a monoclonal antibody (mAb) directed against caspase-3.

Figure 4(c) (activation of QCASP4.1 and QCASP5.1 by lysates of HME cells. Cells were either untreated (control) or treated with staurosporine 1 *μ*M for 6 h, in the absence or the presence of the caspase-3 inhibitor Z-Asp(OMe)-Gln-Met-Asp(OMe)-CH_2_F (100 *μ*M). R.F.U.: relative fluorescent units. Data are mean ± SEM (*n* = 4). **P < .01 versus control and ^††^ *P* < .01 versus staurosporine alone by repeated measures ANOVA) shows that activation of QCASP4.1 and of QCASP5.1 by lysates obtained from staurosporine-treated cells was significantly higher compared to control lysates (at 6 h: for QCASP4.1: control 298 ± 26; staurosporine 573 ± 17 R.F.U. × 10^2^; *P* < .01; for QCASP5.1: control 419 ± 43; staurosporine 886 ± 19 R.F.U. × 10^2^; *P* < .01). This increased fluorescence was abolished by the caspase-3 inhibitor Z-Asp(OMe)-Gln-Met-Asp(OMe)-CH_2_F (QCASP4.1: 146 ± 23 R.F.U. × 10^2^; *P* < .01 versus staurosporine alone; QCASP5.1: 189 ± 30 R.F.U. × 10^2^; *P* < .01 versus staurosporine alone). 

**Figure 5:**
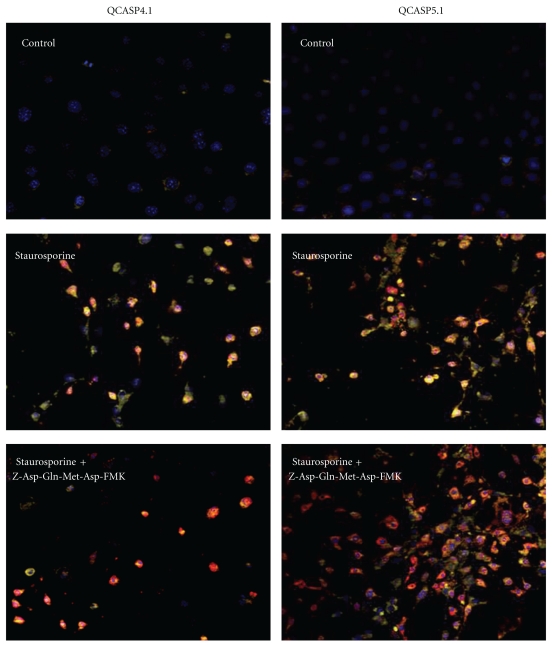
Colocalization of activated QASP4.1- and QCASP5.1-induced fluorescence (yellow) with caspase-3 IF (red) in HME cells. Cells were either untreated (control) or treated with staurosporine 1 *μ*M for 6 h, in the absence or the presence of the caspase-3 inhibitor Z-Asp(OMe)-Gln-Met-Asp(OMe)-CH_2_F (100 *μ*M). Nuclei are stained with Hoechst and appear blue. The orange staining represents the colocalization of QCASP4.1 or QCASP5.1 (yellow) with caspase-3 mAb (red; magnification x20).

Figure 5 shows the staining obtained from cleaved QCASP4.1 and QCASP5.1 (yellow) and from the antiactivated caspase-3 mAb (red). While control cells did not display any detectable activation of probes, in staurosporine-treated cells, the increased positive IF staining for caspase-3 was accompanied by a strong increase in the fluorescence signal emitted by cleaved QCASP4.1 and QCASP5.1 probes. Moreover, in the majority of the cells, we observed a colocalization of caspase-3 mAb and cleaved QCASP4.1 and QCASP5.1 staining. Quantitative analysis showed that 94 ± 3% (*n* = 7) and 74 ± 6% (*n* = 11) of the cells demonstrated colocalization of caspase-3 with QCASP4.1 and QCASP5.1, respectively. Furthermore, the staining obtained from cleaved probes was markedly reduced by the caspase-3 inhibitor Z-Asp(OMe)-Gln-Met-Asp(OMe)-CH_2_F.

### 3.7. Flow Cytometry Analysis of the Comparison between Annexin V and QCASP4.1 and QCASP5.1 Labeling

**Figure 6:**
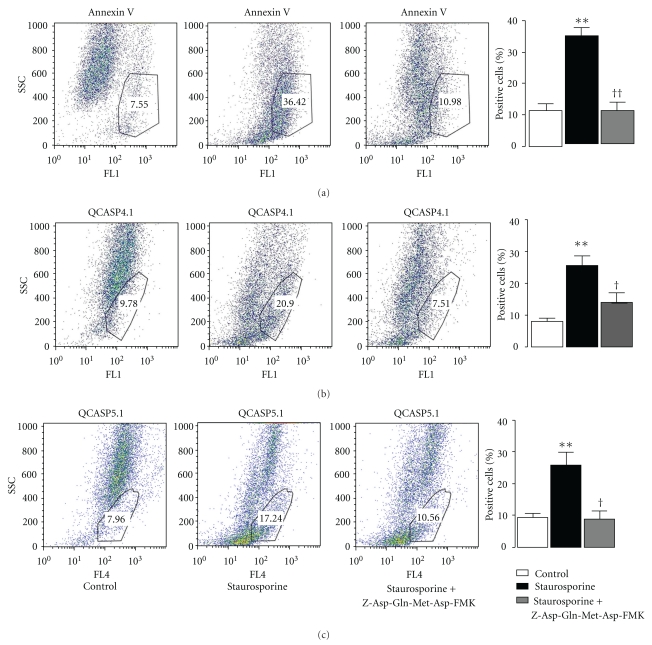
Flow cytometry analysis of Annexin V, QCASP4.1, and QCASP5.1 fluorescence in HME cells. Cells were either untreated (control) or treated with staurosporine 1 *μ*M for 6 h, in the absence or the presence of the caspase-3 inhibitor Z-Asp(OMe)-Gln-Met-Asp(OMe)-CH_2_F (100 *μ*M). **P < .01 versus control; ^†^ *P* < .05,  ^††^ *P* < .01 versus staurosporine alone. Only cells displaying either an FL1 or an FL4 intensity, corresponding respectively to annexin V or QCASP staining, ranging from almost 10² to 10^4^ for both SSC and FSC parameters were included in the analysis gate.

HME cells were incubated with annexin V, QCASP4.1, or QCASP5.1 for 30 min, before analysis. Results are shown in Figure 6. Only 11 ± 5% of control cells were positive for annexin V, corresponding to basal level of apoptosis in normal cell culture. After staurosporine treatment, the number of annexin V positive cells was increased threefold (34 ± 5%; *P* < .01), and this was prevented by pretreatment with the caspase-3 inhibitor Z-Asp(OMe)-Gln-Met-Asp(OMe)-CH_2_F (11 ± 3%). These results with annexin V are quantitatively similar to those obtained for caspase-3 activity (Figure 1), both in terms of the effect of staurosporine and caspase-3 inhibitor. 

Figure 6 also shows that staurosporine significantly increased the number of cells positive for cleaved QCASP4.1 and QCASP5.1 fluorescence, which also increased by about threefold compared to control cells (QCASP4.1: control: 8 ± 3%; staurosporine 26 ± 7%; *P* < .01; QCASP5.1: control: 9 ± 3%; staurosporine 26 ± 10%; *P* < .01), again reinforcing that both probes are being cleaved by apoptotic cells. In addition, the caspase-3 inhibitor significantly reduced the number of staurosporine-treated QCASP4.1 or QCASP5.1-positive cells (QCASP4.1: 14 ± 5%; *P* < .05; QCASP5.1: 9 ± 4%; *P* < .05).

### 3.8. Evaluation of the Capacity of QCASP5.1 to Detect Caspase-3 Activity in an *Ex Vivo* Model of Cardiac Apoptosis

As shown in Figure 7(a) (caspase-3 activity in the left ventricle. Mice were either untreated (control) or treated with an intraperitoneal injection of 20 mg/kg DOX four days before sacrifice. Heart sections from DOX mice were either untreated or treated with the caspase-3 inhibitor Z-Asp-Gln-Met-Asp-FMK. R.L.U.: relative luminescent Units. Data are mean ± SEM (*n* = 6). **P < .01 versus controls; ^††^ *P* < .05 versus DOX alone), a four-day treatment with DOX was associated with a twofold significant increase in left ventricular (LV) caspase-3 activity, which was reduced by the caspase-3 inhibitor Z-Asp(OMe)-Gln-Met-Asp(OMe)-CH_2_F. 

Compared to controls, LV lysates from DOX-treated mice showed a significant increase in the fluorescent signal emitted by cleaved QCASP5.1 (Figure 7(b): activation of QCASP5.1 by LV lysates. Lysates were obtained from mice either untreated (control) or treated with an intraperitoneal injection of 20 mg/kg DOX four days before sacrifice. Lysates from DOX mice were either untreated or treated with the caspase-3 inhibitor Z-Asp(OMe)-Gln-Met-Asp(OMe)-CH_2_F. R.F.U.: relative fluorescent units. Data are mean ± SEM (*n* = 6). **P < .01 versus controls; ^††^ *P* < .01 versus DOX alone). In contrast, this increased cleavage was absent when lysates of DOX mice were incubated with the caspase-3 inhibitor. In parallel, Figure 7(c) (QCASP5.1 fluorescence in LV sections. Sections were obtained from mice either untreated (control) or treated with an intraperitoneal injection of 20 mg/kg DOX four days before sacrifice. Sections from DOX mice were either untreated or treated with the caspase-3 inhibitor Z-Asp(OMe)-Gln-Met-Asp(OMe)-CH_2_F. R.F.U.: relative fluorescent units. Data are mean ± SEM (*n* = 6). **P < .01 versus controls; ^†^ *P* < .05 versus DOX alone) shows that hearts sections obtained from DOX mice and incubated with QCASP5.1 displayed a significant increase in fluorescence, which was also prevented by the caspase-3 inhibitor. Finally, histological examination showed that hearts from DOX-treated hearts displayed a maintained structural integrity and an absence of detectable necrosis (Figure 7(d): typical histological image of an LV section from DOX-treated mice. LV sections were stained with wheat germ agglutine conjugated with fluorescein, and nuclei were counterstained with Bisbenzimide H33342. The scale bar represents a 50-micrometer length.)

## 4. Discussion

The aim of this study was to develop new probes, targeting selectively active caspase-3, for noninvasive optical imaging of apoptosis.

The capacity of the probes to be cleaved selectively by caspase-3 was first evaluated in enzymatic assays using recombinant caspases. The selected panel included enzymes from the three main caspases groups, including inflammatory caspases (1 and 11), initiator caspases (8), and effector caspases (3, 6, and 7) [25]. Importantly, these three latter enzymes are known to recognize the Asp-Glu-Val-Asp sequence incorporated in the structure of the three probes [26]. We found that none of the probes were cleaved by caspases 1, 6, 8, and 11, but to be markedly cleaved by caspase-3 and, to a very small extent, by caspase-7. This clearly shows a very strong selectivity of the probes for caspase-3 *in vitro*, although one cannot fully exclude the possible contribution of other caspases, especially caspase-7.

In order to test for the *in vitro* biological activity of the probes, we first developed a cellular model of apoptosis by using staurosporine. Both flow cytometry and immunofluorescence results demonstrated the apoptotic effect of staurosporine and its ability to activate caspase-3 in HME cells. These results are in agreement with data reported previously in various cell types [27–29]. They suggest that this cell model is appropriate for the validation of probes directed toward caspase-3.

Using this model, we found that lysates from apoptotic endothelial cells efficiently activated the three probes, leading to increased fluorescence from the cleaved probe. Moreover, this increased fluorescence was strongly reduced in the presence of a caspase-3 inhibitor, suggesting that it reflects selective cleavage of the probes by caspase-3. In parallel, we found a strong colocalization of the fluorescent signal with that originating from an antibody recognizing active caspase-3. This suggests that the increased probe fluorescence selectively originates from cells with activated caspase-3.

Surprisingly, the caspase-3 inhibitor did not completely reduce the signal originating from the antibody directed against caspase-3 activity. This persistence in a context of an abolition of the increased activity may be explained by the epitope of mAb against activated caspase-3, which recognizes the terminal residues adjacent to Asp175 that is not located directly in the enzymatic site of the protein, leading to residual mAb binding even in the presence of blocked activity [30, 31]. 

The first generation probe, QCASP3.2, demonstrated a satisfactory capacity to be cleaved and to become fluorescent in the presence of activated caspase-3 (either recombinant or endogenous); however, this probe resulted in a poor cell labeling in flow cytometry experiments (data not shown). Since this was most likely due to insufficient internalization of the probe into cells, our next objective was to improve the capacity of the probes to penetrate into cells, by adding an *α*-helix polyarginine CPP (in QCASP4.1 and QCASP5.1 probes). Moreover, since incorporation of a PEG spacer to peptide- or polymer-based architectures significantly increases circulation times [32, 33], we decided to incorporate a PEG moiety additionally to the CPP (in QCASP5.1 probe).

We first demonstrated that the addition of CPP improved cellular penetration for both probes, although this was not as pronounced with QCASP5.1, compared to QCASP4.1, presumably reflecting the fact that the PEG moiety hampered cell penetration. The lower cell penetration of QCASP5.1, compared to QCASP4.1 may also explain at least in part the lower colocalization with caspase-3, as mentioned in the results. It must be noted, however, that these experiments were successful only when performed in nonapoptotic cells. Indeed, the same experiment performed in staurosporine-treated cells gave inconsistent results because a large part of the apoptotic cells did not adhere to the culture dish, rendering the separation between cells and medium almost impossible. The differences in cell penetration cannot be due to different binding to the cell membrane, since cell cultures are trypsinized and washed before being lysed and incubated with recombinant caspase-3 [34, 35].

As anticipated from the differences in cell penetration, unlike QCASP3.2, the second-generation probes gave satisfactory results in flow cytometry, as demonstrated by the marked increase in the percentage of positive cells after treatment with staurosporine, and the abolition of this effect by the selective caspase-3 inhibitor.

We also compared the ability of QCASP4.1 and QCASP5.1 to detect apoptosis by flow cytometry with that of annexin V. Although it may be limited by unspecific effects [13], annexin V is still a widely used and standard marker of early apoptosis [36], since it is a natural ligand of PS which is translocated at the surface of apoptotic cells. This mechanism occurs upon procaspase-3 activation [37]. These results with annexin V are quantitatively similar to those obtained for caspase-3 activity, both in terms of the effect of staurosporine and caspase-3 inhibitor. It must be noted that these similar quantitative data were obtained in a relatively pure model of apoptosis, thus this does not exclude the possibility that annexin V may overestimate apoptotic cell death in other more integrated models, especially in the presence of mixed apoptotic and necrotic cell death.

Based on these positive results obtained in vitro, our next step was to assess the capacity of the second-generation probes to detect apoptosis *ex vivo*, in a pathologically relevant model of cardiac apoptosis. For this purpose, cardiac apoptosis was induced *in vivo* by doxorubicin (DOX). DOX is an anthracycline commonly used in cancer chemotherapy, and is well known for its capacity to induce cardiac oxidative stress and apoptosis [23], leading to cardiomyopathy and heart failure. Indeed, we confirmed that DOX was associated with an increased caspase-3 activity, suggesting that this model is appropriate for the evaluation of the probes.

We first measured the capacity of LV lysates to cleave probes *ex vivo*. These results demonstrate the ability of both probes to detect apoptosis *via* a caspase-3 mechanism; however, this cleavage appeared much less pronounced with QCASP4.1 than with QCASP5.1. As a result, we failed to detect a significant signal originating from cleaved QCASP4.1 in cardiac tissue sections. Thus, only the data obtained with QCASP5.1 are shown. 

We finally found that cardiac apoptosis (induced by DOX), was associated with a significant increase in fluorescence originating from cleaved QCASP5.1, which was also prevented by the caspase-3 inhibitor, and which paralleled the increased caspase-3 activity. Thus, taken together, these results suggest that QCASP5.1 may efficiently and selectively detect caspase-3 activity *ex vivo* in a clinically relevant model of cardiac apoptosis.

## 5. Conclusion

In this study, we successfully validated cleavable optical bioprobes that can be selectively activated by caspase-3 in both *in vitro* and *ex vivo* models of cardiovascular apoptosis. They thus may form new original tools for the detection of apoptosis not only in cardiovascular diseases but also possibly in cancer or immunoinflammatory diseases.

##  Conflict of Interests

This paper was supported in part by a grant from QUIDD company. C. Portal, B. Delest, H. Ligeret, P. Noack, and M. Massonneau are QUIDD employees.

## Figures and Tables

**Figure 7:**